# Comparative Study of Total Hydroperoxides and Antioxidant Defense System in the Indian Tropical Tasar Silkworm, *Antheraea mylitta*, in Diapausing and Non-Diapausing Generations

**DOI:** 10.1673/031.013.12301

**Published:** 2013-11-05

**Authors:** Karmabeer Jena, Prasanta K. Kar, Chittithoti S. Babu, Shantakar Giri, Shyam S. Singh, Bhagwan C. Prasad

**Affiliations:** Central Tasar Research and Training Institute, Piska Nagri, Ranchi, Jharkhand, India

**Keywords:** antioxidant defense, diapause, oxidative stress, tasar silkworm

## Abstract

In the present study, the total hydroperoxides, catalase, glutathione-s-transferase, and ascorbic acid contents were determined in different developmental stages of the non-diapause and the diapause generation of the tropical tasar silkworm, *Antheraea mylitta* Drury (Lepidoptera: Saturniidae). The results showed stage-specific significantly higher levels of total hydroperoxides, catalase, and ascorbic acid contents in the non-diapause as compared to the diapause generation (*p* < 0.05). However, a significantly enhanced level of glutathione-S-transferase activity was observed in mature 5th instar larvae of the diapause generation (*p* < 0.05). In the case of pupae, significantly higher levels of total hydroperoxides, catalase, and glutathione-s-transferase activity were observed in the non-diapause generation (*p* < 0.05). These results could be the effect of intensive metabolic transformation that takes place in tissues of the non-diapause generation and causes increased production of reactive oxygen species, such as hydroperoxides. The results suggest that antioxidants play an important role in protecting cells against reactive oxygen species.

## Introduction

Reactive oxygen species, such as superoxide radical (O_2_^•-^), hydrogen peroxide, and hydroxyl radical (OH^•^) are generated in aerobic organisms under normal metabolism and when exposed to various abiotic and biotic factors during life spans (Halliwell and Gutteridge 2001). Reactive oxygen species cause oxidative stress leading to the damage of biomolecules such as proteins, lipids, and nucleic acids, resulting in disturbance of homeostasis and cellular death if not eliminated (Hermes-Lima and Zenteno-Savin 2002). Cells have interdependent antioxidant defense mechanisms that protect against damage from oxidative stress.

Insects posses an antioxidant defense system that consists of both enzymatic and nonenzymatic components. The enzymatic components are superoxide dismutase, catalase, glutathione peroxidase, glutathione reductase, and glutathione-S-transferase. O_2_^•-^ radicals are dismutated by superoxide dismutase to hydrogen peroxide, which is reduced to water and molecular oxygen by catalase or neutralized by glutathione peroxidase. This process catalyzes the reduction of hydrogen peroxide to water and organic peroxide to alcohols using reduced glutathione as a source of reducing equivalent. Glutathione reductase regenerates reduced glutathione from oxidized glutathione, which scavenges reactive oxygen species and acts as a substrate for the other enzymes. Glutathione-S-transferase conjugates xenobiotics with reduced glutathione for excretion. The non-enzymatic components consist of small organic molecules such as reduced glutathione and vitamin C (Krishnan and Kodrík 2006; Krishnan et al. 2009; Zhao and Shi 2009, 2010; Buyukguzel et al. 2010). The tropical tasar silkworm, *Antheraea mylitta* Drury (Lepidoptera: Saturniidae), produces silk of commercial importance. Farmers generally prefer rearing this species on *Terminalia tomentosa* Roxburgh (Myrtales: Combretaceae). In tropical India, the life cycle of *A. mylitta* begins in late June with the onset of monsoon season. The first life cycle is completed within 28 to 32 days, which includes the period from the hatching of 1st instar larvae to the formation of the pupa inside the cocoon. In this generation, the pupae behave as non-diapausing, and the next active phase of the life cycle begins with the emergence of moths from the cocoons from late August to early September. The larvae in this phase of the life cycle metamorph into diapausing pupae. The larval period in the diapausing generation is comparatively longer (39–45 days) than that of the non-diapause generation. In earlier studies, it was reported that *A. mylitta* larval growth rate is faster in the non-diapausing generation than in the diapausing one (Mishra et al. 2007; Dinesh Kumar et al. 2008).

Among the silkworm species, antioxidant defense systems have been studied in the mulberry silkworm, *Bombyx mori.* Zhao and Shi (2009) observed differences in antioxidant enzymes (superoxide dismutase, catalase, and xanthin oxidase) and hydrogen peroxide levels in univoltine and polyvoltine strains of *B. mori*. They also studied the effect of chilling on the status of hydrogen peroxide and the activities of superoxide dismutase, catalase, and xanthin oxidase of diapause and non-diapause eggs (Zhao and Shi 2010). Variation in the antioxidant defense in the mitochondria of the European corn borer, *Ostrinia nubilalis*, has been studied in diapause and post-diapause stages (Jovanovic-Galovic et al. 2007). Krishnan et al. (2002) observed high superoxide dismutase activity in hemolymph of *B. mori* due to bacterial infection. However, there is no information on the relationship of prooxidants and antioxidants in diapause and non-diapause generations of *A. mylitta* that may have roles in metabolic processes during larval and pupal development.

Therefore, the present study was undertaken in order to investigate the levels of total hydroperoxides and antioxidant defense in different larval stages and pupae of diapause and non-diapause generations of *A. mylitta*.

## Materials and Methods

### Experimental insects and their maintenance

The bivoltine Daba ecorace of *A. mylitta* was selected for this study. The rearing was conducted on *T. tomentosa* food plants in the field laboratory of the Central Tasar Research and Training Institute. The non-diapausing and diapausing generations of tasar silkworm were reared July through August and September through November respectively.

### Sample preparation

For biochemical assays, 30, 15, 10, 10, and 10 larvae of 1^st^, 2^nd^, 3^rd^, 4^th^, and 5^th^ instar larvae were taken from both non-diapausing and diapausing generations, respectively. Similarly, 10 pupae of both sexes were taken from both generations. For sample preparation, 5 homogenates were maintained in each case. Tissue samples (fat body and whole body) were thoroughly washed, surface dried on filter paper, and homogenized with 50 mM phosphate buffer (pH 7.0). Homogenization was carried out at 4° C in a motor driven Teflon Potter-Elvehjem homogenizer with 12–15 strokes, filtered through cheese cloth, and centrifuged at 8000 × g for 15 min at 4° C. The supernatant was used for biochemical analysis.

### Estimation of total hydroperoxides

Hydroperoxides were determined spectrophotometrically according to the method of ferrous oxidation with xylenol orange (Wolff 1994). The reaction mixture contained 100 µM xylenol orange, 250 µM ammonium ferrous sulphate, 100 mM sorbitol, 25 mM H_2_SO_4_, and the sample. The absorbance was read at 560 nm after centrifugation at 4000 × g for 10 min. The signal was read against hydrogen peroxide standard curve, and the unit was expressed as µM/mg protein.

### Catalase activity

Catalase activity was determined according to the method of Aebi (1974) on the basis of the decomposition rate of hydrogen peroxide by the enzyme. The assay mixture contained 20 mM hydrogen peroxide and the sample (100 µg protein). Absorbance was measured at 240 nm, and catalase activity was expressed as nkat/mg protein (1 katal = 1 mol sec^-1^).

### Glutathione-S-transferase activity

Glutathione-S-transferase activity was measured according to Habig et al. (1974) using 1-chloro-2, 4-dinitrobenzene (CDNB) as a substrate. The assay mixture contained 100 mM phosphate buffer (pH 7.0), 30 mM reduced glutathione, 15 mM CDNB, and the sample (100 µg protein). The change in absorbance was recorded at 340 nm, and enzyme activity was expressed as nmol CDNB conjugate formed/min/mg protein using a molar extinction coefficient of 9.6 mM^-1^ cm^-1^.

### Estimation of ascorbic acid

Samples were precipitated in 5% (w/v) trichloroacetic acid in an ice bath and centrifuged at 5000 × g for 10 min. The deproteinized supernatant was used for the estimation of ascorbic acid according to the method of Mitusi and Ohata (1961). The reaction mixture contained 2% sodium molybdate, 0.15 N H_2_SO_4_, 1.5 mM Na_2_HPO_4_, and the sample. The mixture was boiled at 90° C for 45 min and then centrifuged at 1000 × g for 15 min. The absorbance of the supernatant was measured at 660 nm. Ascorbic acid was taken as the standard and results were expressed as µg /mg protein.

Protein content was estimated by the Folin—Phenol method of Lowry et al. (1951), using bovine serum albumin as the standard.

### Statistical analysis

Two-way analysis of variance (ANOVA) was used to compare the mean values among different larval instars and pupae, and the nondiapausing and diapausing generations were kept as independent variables. ANOVA was done separately for the 1^st^ to 3rd instar group, the 4^th^ to 5^th^ instar group, and the male and female pupae groups due to the differences in sample preparation. The mean values of each stage of the non-diapausing and diapause generations were compared by using Student's *t*test. Discriminate function analysis was carried out to test the veracity of the distribution of the two larval groups with SPSS 11.5 (SPSS Inc.). The distribution of the different groups was shown on a two-dimensional canonical correlation plot.

## Results

### Total hydroperoxides

**Figure 1.**
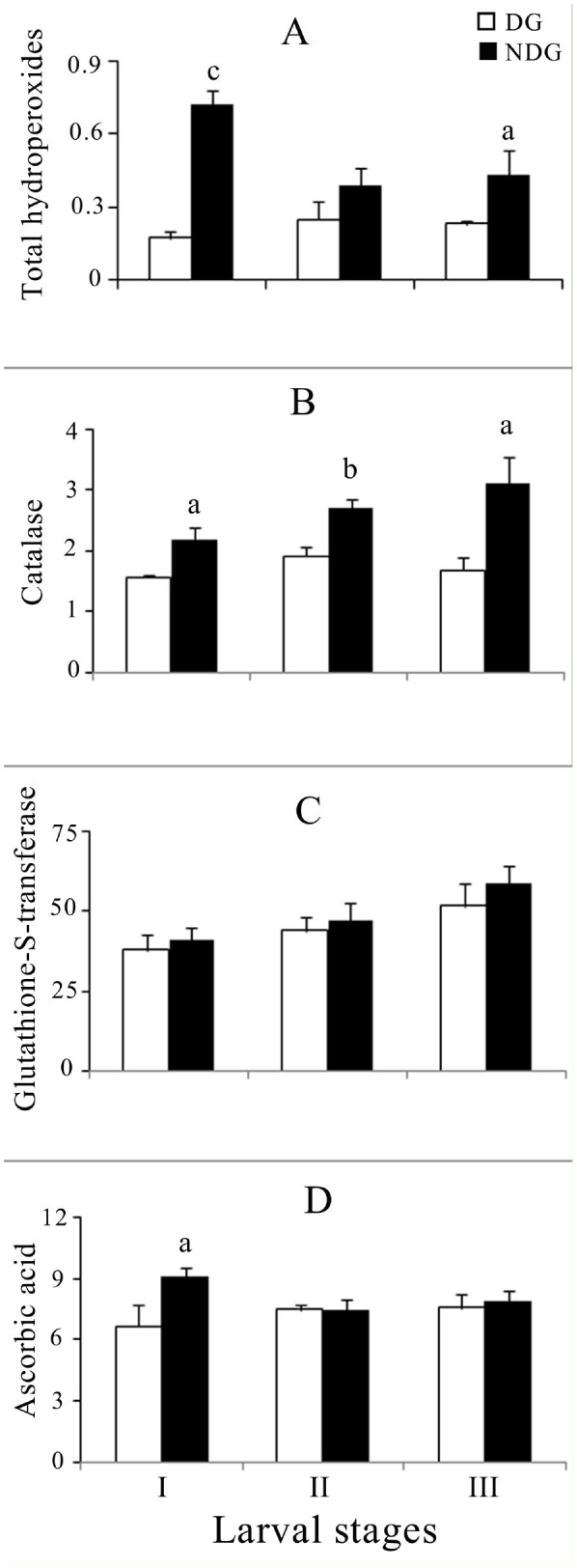
(A) Variation of total hydroperoxide (µM H_2_O_2_/mg protein), (B) catalase (µKat/mg protein), (C) glutathione-Stransferase (nmol CDNB conjugate formed/min/mg protein), (D) ascorbic acid content (µg ASA/mg protein) in diapause and non-diapause *Antheraea mylitta* 1^st^ to 3^rd^ instar larvae. Data are expressed as mean ± SEM (n = 5). Superscript letters indicate a significant difference between diapause and non-diapause: ^a^ *p* < 0.05, ^b^ *p* < 0.01, ^c^ *p* < 0.001. High quality figures are available Online.

**Figure 2.**
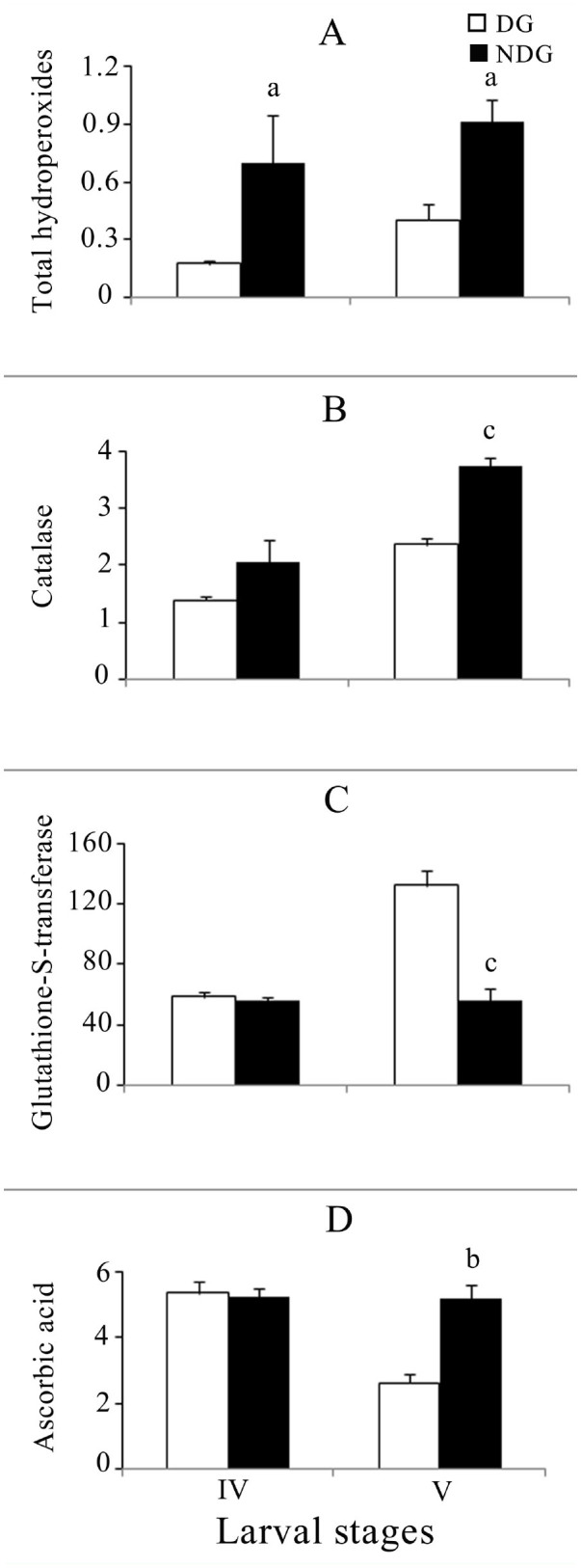
(A) Variation of total hydroperoxide (µM H_2_O_2_/mg protein), (B) catalase (µKat/mg protein), (C) glutathione-Stransferase (nmol CDNB conjugate formed/min/mg protein), (D) ascorbic acid content (µg ASA/mg protein) in diapause and non-diapause *Antheraea mylitta* 4^th^ and 5^th^ instar larvae. Data expressed as mean ± SEM (n = 5). Superscript letters indicate a significant difference between diapause and non-diapause: ^a^ *p* < 0.05, ^b^ *p* < 0.01, ^c^ *p* < 0.001. High quality figures are available online.

**Figure 3.**
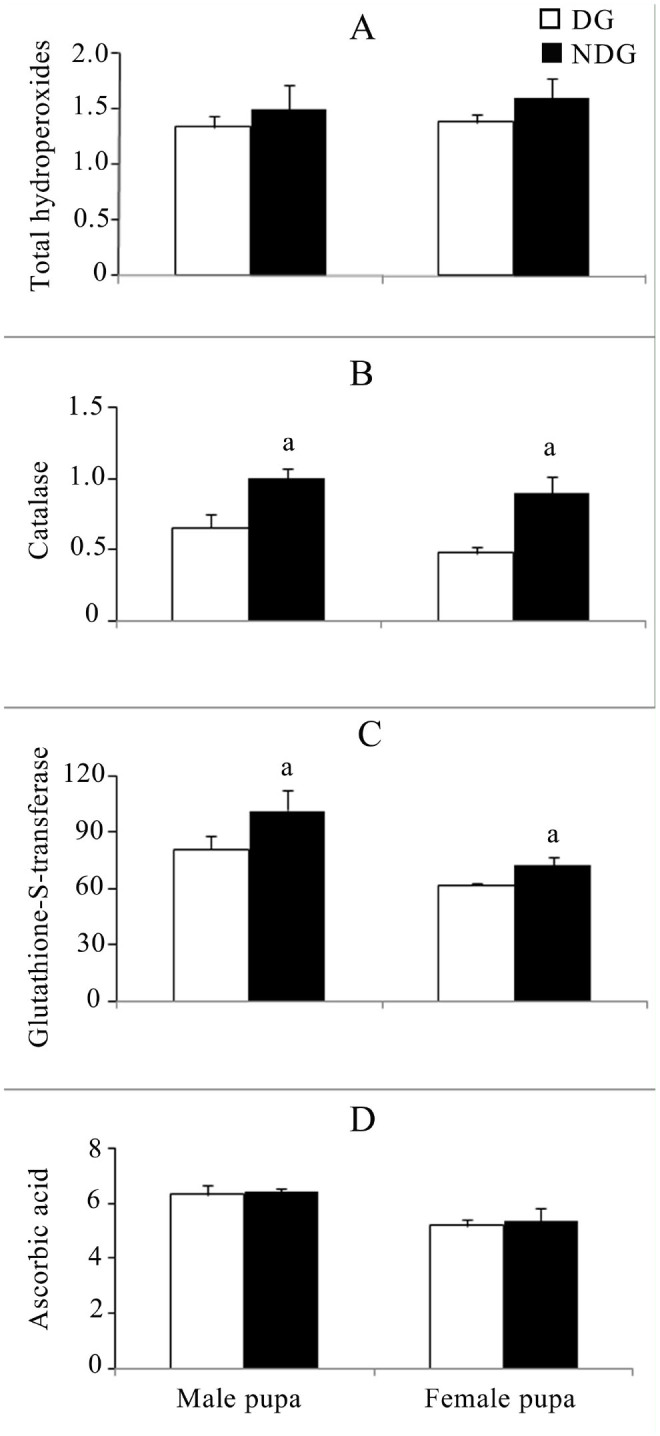
(A) Variation of total hydroperoxide (µM H_2_O_2_/mg protein), (B) catalase (µKat/mg protein), (C) glutathione-Stransferase (nmol CDNB conjugate formed/min/mg protein), (D) ascorbic acid content (µg ASA/mg protein) in diapause and non-diapause *Antheraea mylitta* pupae. Data expressed as mean ± SEM (n = 5). Superscript letter indicates a significant difference between diapause and non-diapause: ^a^ *p* < 0.05. High quality figures are available online.

A stage-specific significant variation of total hydroperoxides levels was observed among the diapause and non-diapausing generation larvae. Significant differences were seen between diapausing and non-diapausing generations in all instars except the 2^nd^ (Figures 1A and 2A). ANOVA revealed highly significant variation between diapause and non-diapause generations in both 1^st^ to 3^rd^ (F = 38.259, *p* < 0.001) and 4^th^ to 5^th^ (F = 15.99, *p* < 0.001) instar larval groups. Although there was no significant variation among them, the interaction between diapause and larval stage was significant (F = 6.803, *p* < 0.01). In male and female pupae, the mean level of total hydroperoxides was found to be insignificant in both diapause and nondiapausing generations (Figure 3A).

### Catalase

Catalase activity was higher in the nondiapausing generation than in the diapausing generation (Figure 1B and 2B). ANOVA showed highly significant differences between the diapausing and non-diapausing generations (1^st^ to 3^rd^ instar: F = 30.288, *p* < 0.001; 4^th^ to 5^th^ instar: F = 5.26, *p* < 0.05) and the different larval stages (1^st^ to 3^rd^ instar: F = 3.55, *p* < 0.05; 4^th^ to 5^th^ instar: F = 15.05, *p* < 0.01). In the non-diapausing generation, the corresponding levels of catalase activities were statistically significant for both males (*p* < 0.05) and females (*p* < 0.05) (Figure 3B). The variation was significant only between generations (F = 27.209, *p* < 0.001).

### Glutathione-S-transferase

Glutathione-S-transferase activity increased in the diapause generation in 1^st^ to 5^th^ instars. In the non-diapausing generation larvae, enzyme activity was also increased from 41.25 to 56.468 nmol CDNB conjugate formed/min/mg protein in the 1^st^ to 5^th^ instars (Figure 1C, 2C). Significant differences were seen between diapausing and non-diapausing generations only in the 5th instar (Figure 2C). ANOVA revealed significant variation among larval instars in both 1^st^ to 3^rd^ (F = 5.58, *p* < 0.01) and 4^th^ to 5^th^ (F = 38.84, *p* < 0.001) larval groups. In the case of the 4^th^ and 5^th^ instars, significant variance (F = 43.96, *p* < was observed between the diapause and non-diapause generation. In the nondiapausing generation, the corresponding enzyme activity was statistically significant for both males (*p* < 0.05) and females (*p* < 0.05) (Figure 3C). The variation was significant between both generations (F = 6.48, *p* < 0.05) and sexes (F = 15.69, *p* < 0.01).

### Ascorbic acid

In the diapause generation larvae, ascorbic acid levels were similar in the 1^st^ to 3^rd^ instars and declined in the 4^th^ and 5^th^ instars (Figures 1D and 2D). Significant differences were seen between diapausing and non-diapausing generations only in the 1st and 5th instars. ANOVA results of the 4^th^ and 5^th^ instar revealed significant variation between generations (F = 14.57, *p* < 0.01) and larval instars (F = 20.21, *p* < 0.001). In the nondiapause and diapause generation pupae, ascorbic acid contents were 6.39 ± 0.152, 6.32 ± 0.31, 5.34 ± 0.46, and 5.19 ± 0.23 µg / mg protein in males and females respectively (Figure 3D). ANOVA revealed significant variation between sexes alone (F = 15.12, *p* < 0.01).

### Discriminant function analysis and canonical correlation

**Figure 4.**
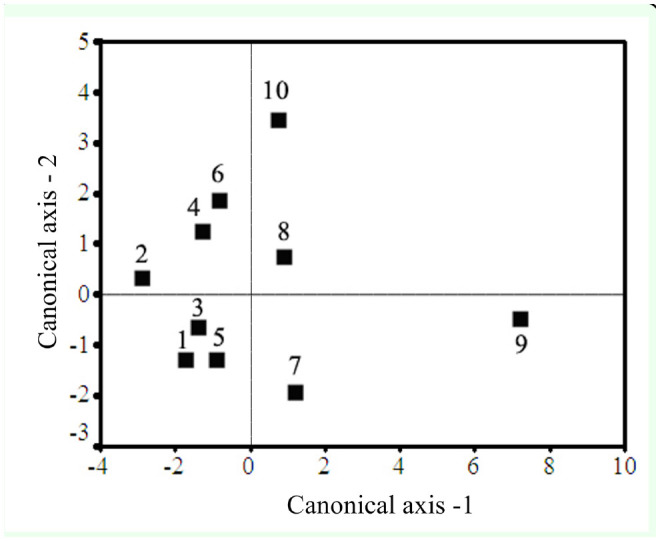
The canonical discriminant space showing the centroids of different groups. 1, 3, 5, 7, and 9 indicate diapausing *Antheraea mylitta* larvae of 1^st^ to 5^th^ instar, respectively. 2, 4, 6, 8, and 10 indicate non-diapausing larval instars from 1^st^ to 5^th^ instar, respectively. High quality figures are available online.

**Figure 5.**
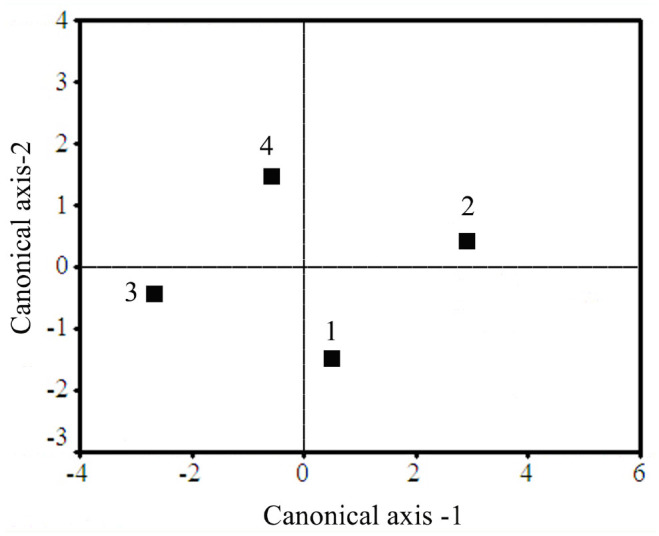
The canonical discriminant space showing the centroids of different groups. 1 and 3 represent the male and female *Antheraea mylitta* pupae of the diapausing generation, and 2 and 4 represent the male and female pupae of the nondiapausing generation. High quality figures are available online.

Discriminate function analysis was performed for all the larval stages of the diapausing and non-diapausing generations (Figure 4). In all larval stages, 86.8% of variance was explained in the first two functions with Eigen values of 9.792 and 2.944 for the first and second function respectively, with highly significant canonical correlations of 0.953 and 0.864 respectively. Wilk's lambda value was 0.006 with a χ^2^ value of 214.279 (*p* < 0.001). In the case of the pupae, 99.5% of variances were explained with Eigen values of 4.798 and 1.454 for the first and second function respectively, with highly significant canonical correlations of 0.910 and 0.770 respectively. Wilk's lambda value was 0.068 with a χ^2^ value of 40.295 (*p* < 0.001). For the larvae, the non-diapausing and diapausing generations were grouped separately. There was a clear difference between male and female pupae in relation to overall oxidant and antioxidant status in diapausing and non-diapausing generations (Figure 5).

## Discussion

Earlier studies on the reactive oxygen species and antioxidant defense mechanism in insects suggested that there exists a regulatory mechanism for balancing prooxidants and antioxidants (Ahmad 1992). In similar studies, prooxidant and antioxidant balance was shown to affect development and aging in *Drosophila melanogaster* (Klichko et al. 2004). Reactive oxygen species modulate immunity against pathogens (Molina-Cruz et al. 2008), and catalase affects fecundity in *Anopheles gambiae* (Dejong et al. 2007). The tasar silkworm *A. mylitta* has been studied for its growth and development in non-diapausing and diapause generations (Mishra et al. 2007; Dinesh Kumar et al. 2008), but to date there is no information on the antioxidant defense system in *A. mylitta* related to its endogenous or exogenous sources of reactive oxygen species and the balancing role of enzymatic and non-enzymatic antioxidants. The findings of the present study indicate higher levels of total hydroperoxides in larvae and pupae of the non-diapausing generation of *A. mylitta* (Figure 1A, 2A, 3A). Loft et al. (1994) reported that higher respiration is linked with higher production of reactive oxygen species. The elevated level of total hydroperoxide in the non-diapausing generation of *A. mylitta* can be explained by the fact that oxygen consumption is higher during this generation than it is in the diapausing generation (Rath et al. 2005). *A. mylitta* shows a higher growth rate and a shorter larval period in the nondiapause and vice versa in the diapause generation (Mishra et al. 2007; Dinesh Kumar et al. 2008). Insect diapause is tuned to the slow utilization of existing energy reserves, resulting in a lower rate of respiration and a lower content of mitochondrial cytochrome and activation of respiratory enzymes (Jovanovic-Galovic et al. 2004 and references therein), which might have been responsible for the lower-level production of peroxides in diapausing *A. mylitta*.

Among antioxidant enzymes, catalase is the primary scavenger of hydrogen peroxide in the cell. The importance of this enzyme lies within the Fenton reaction (Fe(II) / Cu(I) + H_2_O_2_ 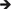 HO^•^+ HO^-^+ Fe(III) / Cu(II); Halliwell and Gutteridge 2001). Increased catalase activity detected in the larvae and corresponding pupae of the non-diapausing generation (Figure 1B, 2B, 3B) could be due to protection of the cells against hydrogen peroxide. Higher growth rate (Mishra et al. 2007; Dinesh Kumar et al. 2008) and higher oxygen consumption (Rath et al. 2005) might have resulted in increased reactive oxygen species production, such as hydrogen peroxide (Figure 1A, 2A, 3A). Elevated levels of hydrogen peroxide might have resulted in the induction of catalase activity, ultimately resulting in neutralization by conversion to water. The higher level of catalase activity was also observed in non-diapause larvae and corresponding pupae of the European corn borer, *Ostrinia nubilalis*, which corroborates with our findings (Jovanovic-Galovic et al. 2004).

Glutathione-S-transferases are a group of detoxification enzymes mainly localized in the cytosol that catalyze the conjugation of reduced glutathione with a wide range of metabolites bearing electrophilic sites (Habig et al. 1974). An increased glutathione-Stransferase activity in larvae of the diapausing generation was observed in the present study (Figure 2C). Earlier reports suggested that the ingestion of prooxidant rich food increases the glutathione-S-transferase activity (Peri- Mataruga et al. 1997). A higher level of phenolic content was observed in *T. tomentosa* during October to December (Sharan et al. 2005), which coincides with the feeding period of diapausing larval generations. The difference observed can be explained by the consumption of higher phenolic compounds by silkworms during that period. However, further studies would be needed to characterize the natural variability of glutathione-S-transferase isoenzyme in relation to abiotic and biotic factors.

Ascorbic acid performs various important physiological functions, such as collagen synthesis and formation of connective tissues (Wilson and Poe 1973), and is a direct scavenger of reactive oxygen species (Halliwell and Gutteridge 2001). In the present study, a stage-specific higher level of ascorbic acid was detected in non-diapausing generation larvae (Figure 1D, 2D). Many reports are not available on the diapause and non-diapause variation of ascorbic acid in *A. mylitta*. The increased level of ascorbic acid might be related to dietary intake or it may be an adaptive mechanism of cells/tissues to cope with oxidative stress. Further studies are required to understand the exact function of ascorbic acid in diapausing and nondiapausing generations.

Rath et al. (2005) reported that the rate of oxygen intake in diapausing *A. mylitta* pupae was lower than in non-diapausing pupae. Similar observations were also made by Satapathy and Mittra (2001), who showed that *A. mylitta* with one generation per year (univoltine) had the lowest metabolic rate in comparison to the insect having three generations per year (trivoltine), as derived from the rate of oxygen consumption. The metabolism remains at the minimum during the diapausing stage of insects (Shappirio 1974a, b). Similarly, it was found that the larval period of non-dispause was very fast (28 days) in comparison to diapause generation larvae (39 days) (Mishra et al. 2007), indicating rapid metabolism during the non-diapause period. Because of its higher metabolism, there are more reactive oxygen species and more antioxidants in non-diapause stages.

A comparison study of peroxides and antioxidant defense in diapause and non-diapause *A. mylitta* showed that the elevation of all the oxidative and antioxidative components (with the exception of glutathione-S-transferase activity in 5th instar larvae) occurred in the non-diapausing generation. These components may provide adequate protection against oxidative stress. Zhao and Shi (2009) observed the variation of antioxidant enzymes (superoxide dismutase, catalase, and xanthin oxidase) and hydrogen peroxide levels in univoltine and polyvoltine strains. They noticed that the metabolism of hydrogen peroxide exhibited significant differences between univoltine and polyvoltine strains and between embryonic and pupal stages. Recently, Zhao and Shi (2010) studied the effect of chilling on the hatchability and the metabolism of hydrogen peroxide, as well as the activities of superoxide dismutase, catalase, and xanthin oxidase of diapause and non-diapause eggs. In *A. mylitta*, the adults are non-feeding, and the larvae cease food intake at the end of the 5^th^ instar. All the activities related to food intake and metabolism are completed in the larval stages before the metamorphosis into pupae, and the 5^th^ instar is the most vital stage for storage of metabolic reserves for all activities in the subsequent life cycle stages.

From the above study it can be concluded that there is a very interesting role of the balance between reactive oxygen species and antioxidants in different life cycle stages as well as diapausing and non-dispausing of *A. mylitta*. There were clear differences in the expression of enzymes in the larval stages of diapausing and non-diapausing generation. The timing and regulatory processes involved in maintaining the balance between oxidants and antioxidants need further study.

## Abbreviations

**CDNB**,: 1-chloro-2, 4-dinitrobenzene
